# Editorial: Bioassays for Assessing Traditional Medicines: Promises and Pitfalls

**DOI:** 10.3389/fphar.2022.839988

**Published:** 2022-02-11

**Authors:** Zhirui Yang, Dan Yan

**Affiliations:** ^1^ Beijing Institute of Clinical Pharmacy, Beijing Friendship Hospital, Capital Medical University, Beijing, China; ^2^ Beijing Key Laboratory for Evaluation of Rational Drug Use, Beijing Shijitan Hospital, Capital Medical University, Beijing, China

**Keywords:** traditional medicines, bioassay, quality control, methodology development, association with clinic

Traditional medicines (TMs) have made a great contribution to the human healthcare over thousands of years. Therefore, it is of great importance to effectively assess the quality of TMs, which is necessary to ensure their clinical therapeutical effect. With the natural origin, TMs contain a complex chemical composition with numerous unknown components, bringing inevitable difficulties in quality control by chemical approaches (the identification or determination of components). Besides, chemical approaches are insufficient to provide an evidence of the relationship between components and bioactivities. Thus, bioassay, a qualitative or quantitative evaluation of the specific biological effects of tested drugs on biological systems (animals, tissues, organs, microorganisms, and cells, as well as related biological factors, etc.) under specific experimental conditions, is proposed to comprehensively control the quality of TMs. Importantly, the methodology for TM bioassay still needs further development, and some key issues including biochemical principle, experimental system, application scope, and the limitation of bioassays in the quality control of TMs need to be elucidated. Accordingly, this Research Topic serves as a forum to solve the above-mentioned problems by publishing researches on establishing experimental systems, developing novel techniques or data processing models, elucidating mechanisms, or proposing perspectives and so forth, with the purpose of better improvement of the safety and effectiveness of TMs.

Given that TMs have apparent characteristics of multiple components and targets, it is essential to use proper approaches matching with the characteristics of TMs in bioassay. Noteworthy, the application of multiomics has contributed greatly to the overall description of the homeostatic intervention by TMs, markedly coincident with the characteristics of TMs that work as a holistic system in the treatment of diseases. Importantly, the multiomics is potent to reflect the integrative characteristics of both pathophysiological status and pharmacological effect, which plays an essential role as a bridge connecting efficacy indicator, syndrome feature, drug action, and biological mechanism. Hence, the strategy based on multiomics has been paid closed attention due to its strong capability to elucidate the mechanism of the action of TMs and find appropriate candidates for bioassay. Following this strategy, Gao et al. conducted a bioassay for the quality control of Lianhua Qingwen capsule, a traditional Chinese medicine formula used in the treatment of influenza A virus infection. As the formula effectively improved influenza-like symptoms in the mice infected with influenza A virus, it also obviously changed the metabolic profiling, as suggested by the metabolomic study. Using serum metabolomics analysis, prostaglandin F_2α_ and arachidonic acid and their metabolic pathway, arachidonic acid metabolism, were identified as the key feature strongly involved in the treatment of influenza A virus infection by the formula. Consistently, the transcriptomics study highlighted the prominent role of cyclooxygenase-2 as the major rate-limiting enzyme in the arachidonic acid metabolism pathway, of which expressions in the lung tissues were validated by RT-qPCR. More importantly, the discovery of cyclooxygenase-2 as a key target in treating influenza pneumonia with the formula by metabolomics and transcriptomics analysis is successfully applied to the bioassay of the formula. Using a cyclooxygenase-2 inhibitor screening kit, it was observed that the biological potency of denatured samples was remarkably lower than that of commercial samples, which suggested that this multiomics-based bioassay was sensitive to distinguish the quality of different Lianhua Qingwen samples. Associated with therapeutic effect and quality evaluation, this study established a good example of systematic methodology in the bioassay, which is theoretically and commercially profound in the quality control of TMs. Additionally, metabolomics combined with chemometrics could be applied to screen the key component panel as the discriminatory quality markers of TMs. Guo et al. proposed an LC-MS-based metabolomics coupled with chemometrics to distinguish the crude and salt-fired *Eucommiae Cortex*, which came from *Eucommia ulmoides* Oliv. In the family of *Eucommiaceae*. The combinatorial markers filtered by orthogonal partial least-squares discriminant analysis, random forest, and partial least squares regression were eventually quantified, and their ability to differentiate crude and salt-fired *Eucommiae Cortex* was validated by discriminant analysis. Thus, this study established a method in screening discriminatory markers to distinguish different processed TMs. As the different processed TMs exert different biofunctions, this study might provide an insight on the improvement of the specificity of the bioassay. Apart from omics, the classic pharmacological study can also provide direct evidence of the bioactivity of TMs, which forms a solid basis for bioassays. It was demonstrated by Qiu et al. that Buxin Yishen decoction was able to improve heart function and delay the progress of renal fibrosis in the myocardial infarction-induced type 2 cardiorenal syndrome. Several targets, including connective tissue growth factor, α-smooth muscle actin, and low-density lipoprotein receptor-related protein, were strongly involved in the underlying mechanism of Buxin Yishen decoction. Hence, a bioassay for the quality control of Buxin Yishen decoction or other related medical products can be designated based on these key targets in the treatment of type 2 cardiorenal syndrome. Besides, as TMs contain various components with multiple targets, the study on the chemical composition or bioactivity might be time consuming and cost intensive. Thus, researches based on big data retrieval, computer simulation, or virtual screening can be a wonderful assistant during the discovery of the biofunctions of TMs. Guo et al. proposed a clinical prescription-protein-small molecule-disease strategy, namely, the CPSD. It was suggested that the classical prescriptions could be used as the source of potential drugs to screen small molecules that regulate specific targets for the drug development. Exemplified by Linggui Zhugan decoction, a library of chemical components was constructed, and the compound–protein interaction was analyzed by semi-flexible docking, which successfully identified the potential targets of Linggui Zhugan decoction in the treatment of arrhythmia. Conveniently and quickly, such strategy could be an alternative to explore the bioactivity of TMs, providing rich information for the bioassay methodology development.

It is well accepted that TMs are difficult to comprehensively characterize by only chemical approaches because of their diverse components and various targets. Bioassay is considered as a useful approach to understand the properties of TMs with biostatistics as a tool, which would be more suitable for evaluating complex systems like TMs, with more practical value and advantages than conventional chemical approaches. Notably, the improvement of methodology is critical to the development of bioassay. Basically, the design of bioassay should follow the basic principles of pharmacological studies to be random, controlled, and repeatable. Clear background information, well-controlled interferences, sensitive indicators, and low detection cost should be taken into consideration when choosing experimental systems (enzymes, ion channels, receptors, subcellular organelles, cells, tissues, microorganisms, organs, and animals, etc.) in bioassays. The detection of biopotency using reasonable group sets of different dosages is recommended in bioassays, as well as the negative control and the positive control with similar mechanisms for better experiment system reliability and stability. To be subjective, clear, and specific, the indicators selected in bioassays should have rationality and representativeness, with closed relationship to the clinical efficacy or safety of TMs. The selection of qualitative, semi-quantitative, and quantitative techniques as well as the suitable biostatistical method is also a key scientific issue in the methodology of TM bioassay. Besides, novel strategies should be proposed for the bioassay methodology establishment of some TMs with opposite clinical efficacy (e.g., *Angelicae Sinensis Radix* from *Angelica sinensis* (Oliv.). Diels with functions of both blood circulation activation and hemostasis when different parts of the plant are used) or those with ambiguous bioactivities (e.g., *Dioscoreae Rhizoma* from *Dioscorea opposita* Thunb.) The studies in this Research Topic undoubtably promote the establishment of bioassay methodology. It has been declared by the US Food and Drug Administration that the biological assay is an essential part of the registration and evaluation of new botanical medicines in the quality control, clinical pharmacology, evidence to ensure therapeutic consistency, and postmarketing consideration, which suggests that the ideology and methodology in the bioassay has achieved a consistency in the development of TMs. With the integration of clinical efficacy confirmation, bioactive component clarification, and underlying mechanism elucidation, the improved bioassay system could promote the modernization and internationalization of TMs, gaining more attention from the world community.

